# Characterizing baseline concentrations, proportions, and processes controlling deposition of river-transported bitumen-associated polycyclic aromatic compounds at a floodplain lake (Slave River Delta, Northwest Territories, Canada)

**DOI:** 10.1007/s10661-016-5277-4

**Published:** 2016-04-12

**Authors:** Matthew C. Elmes, Johan A. Wiklund, Stacey R. Van Opstal, Brent B. Wolfe, Roland I. Hall

**Affiliations:** Department of Geography and Environmental Studies, Wilfrid Laurier University, 75 University Ave West, Waterloo, ON N2L 3C5 Canada; Department of Geography and Environmental Management, University of Waterloo, 200 University Ave West, Waterloo, ON N2L 3G1 Canada; Department of Biology, University of Waterloo, 200 University Avenue West, Waterloo, ON N2L 3G1 Canada

**Keywords:** River monitoring, Polycyclic aromatic compounds, Oil sands, Slave River Delta, Paleolimnology, Pre-industrial baseline

## Abstract

Inadequate knowledge of baseline conditions challenges ability for monitoring programs to detect pollution in rivers, especially where there are natural sources of contaminants. Here, we use paleolimnological data from a flood-prone lake (“SD2”, informal name) in the Slave River Delta (SRD, Canada), ∼500 km downstream of the Alberta oil sands development and the bitumen-rich McMurray Formation to identify baseline concentrations and proportions of “river-transported bitumen-associated indicator polycyclic aromatic compounds” (indicator PACs; Hall et al. 2012) and processes responsible for their deposition. Results show that indicator PACs are deposited in SD2 by Slave River floodwaters in concentrations that are 45 % lower than those in sediments of “PAD31compounds”, a lake upstream in the Athabasca Delta that receives Athabasca River floodwaters. Lower concentrations at SD2 are likely a consequence of sediment retention upstream as well as dilution by sediment influx from the Peace River. In addition, relations with organic matter content reveal that flood events dilute concentrations of indicator PACs in SD2 because the lake receives high-energy floods and the lake sediments are predominantly inorganic. This contrasts with PAD31 where floodwaters increase indicator PAC concentrations in the lake sediments, and concentrations are diluted during low flood influence intervals due to increased deposition of lacustrine organic matter. Results also show no significant differences in concentrations and proportions of indicator PACs between pre- (1967) and post- (1980s and 1990s) oil sands development high flood influence intervals (*t* = 1.188, *P* = 0.279, *d.f.* = 6.136), signifying that they are delivered to the SRD by natural processes. Although we cannot assess potential changes in indicator PACs during the past decade, baseline concentrations and proportions can be used to enhance ongoing monitoring efforts.

## Introduction

**Fig. 1:**
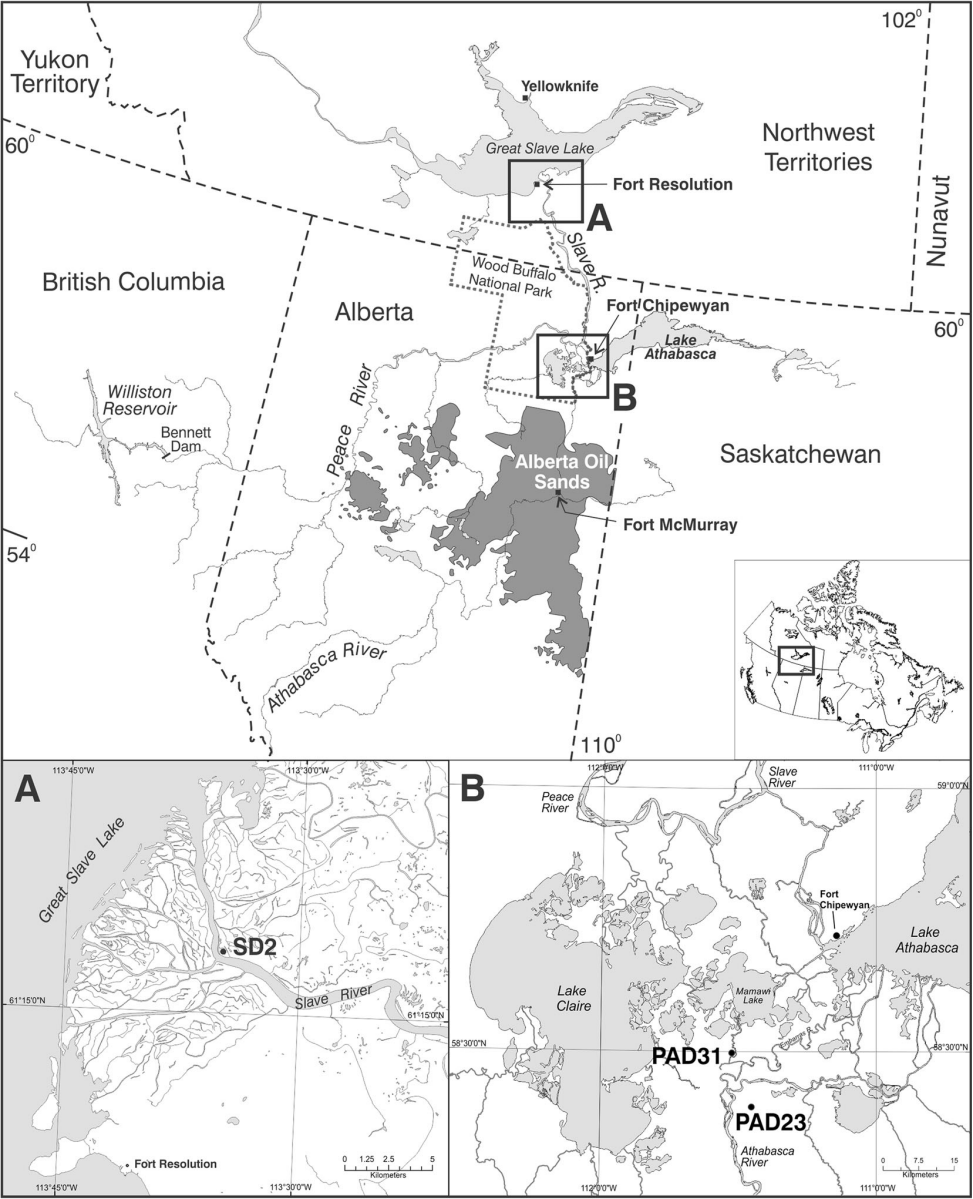
*A*, *B* Location of the study lake, SD2, within the Slave River Delta, located approximately 500 km downstream of the Alberta oil sands development near Fort McMurray. Also shown are lakes PAD31 and PAD23, located in the Athabasca sector of the Peace-Athabasca Delta, which are referred to in the text

The Alberta oil sands are the third largest proven crude oil reserve in the world, after Saudi Arabia and Venezuela (Alberta Energy 2014a). Approximately $159.5 billion was invested in oil sands projects from 2001 to 2012 (Alberta Energy 2014b). In 2012, bitumen production averaged 1.9 million barrels per day, which is expected to grow to 3.8 million barrels per day by 2022. Clearly, oil sands production will continue to be a significant component of Canada’s resource economy and energy security. As oil sands industrial activities have grown, so too have concerns and controversy regarding perceived associated negative effects on environmental and human health (Gosselin et al. 2010). Among the concerns is the potential downstream transport of polycyclic aromatic compounds (PACs) that are contained in the bitumen reserves and potentially released into the environment during mining and processing of the oil sands (Kelly et al. 2009, 2010; Gosselin et al. 2010; Dowdeswell et al. 2010). However, because the Athabasca River and its tributaries flow through and erode strata containing bitumen (Fig. 1), it is necessary to quantify the contribution of naturally sourced contaminant loads to be able to detect pollution (Dowdeswell et al. 2010).

A key challenge lies in determining baseline, pre-disturbance river sediment contaminant concentrations. Current monitoring programs cannot readily distinguish industrial from natural sources of bitumen-associated PACs, because no measurements of river contaminant loads were obtained before industrial development began. To address this key knowledge gap, a paleolimnological approach was applied by Hall et al. (2012) in the Peace-Athabasca Delta (PAD), ∼200 km downstream of oil sands mining activities and where concerns regarding oil sands pollution have been raised (Timoney and Lee 2009; Schindler 2010). Sediment records spanning the past 200 years were utilized to determine pre-industrial concentrations of PACs originating from the Athabasca River and were compared to those values in sediments deposited by floodwaters into one basin (“PAD31”) after the development began in 1967. They used paleohydrological knowledge from prior studies (Wolfe et al. 2008) to identify when the lakes received strong influence from Athabasca River floodwaters. Results demonstrated that sediments deposited before the onset of oil sands development contained PACs eroded from bitumen in the McMurray Formation and transported via the Athabasca River. By comparing PAC composition in sediments deposited in flood-prone and not flood-prone intervals, Hall et al. (2012) identified seven indicator compounds that are common in bitumen and supplied via floodwaters, which they termed “river-transported bitumen-associated indicator PACs” (C2–C4 dibenzothiophenes (D2–D4), C2–C4 fluoranthenes/pyrenes (FLPY2–FLPY4), and C2 sub’d B(a)A/chrysene (BAC-2)). Based on results from PAD31, they found no significant increases in concentrations or proportions of these PACs after the onset of oil sands development.

The paleolimnological results from Hall et al. (2012) were critical for determining baseline concentrations of organic contaminants within the PAD and demonstrating that natural erosion of bitumen has long been a source of associated PAC deposition downstream of the oil sands development. However, it remains unknown how far downstream these bitumen-associated contaminants accumulate. As part of an initiative led by the Slave River and Delta Partnership (http://www.nwtwaterstewardship.ca/node/74), we launched a paleolimnological project to assess the nature of PAC (this study) and metal (MacDonald et al. 2016) deposition in the Slave River Delta (SRD), building on the success of similar studies in the PAD (Hall et al. 2012; Wiklund et al. 2012, 2014). The SRD is located approximately 500 km downstream of the oil sands development, and the Slave River receives about 34 % of its annual discharge from the Athabasca River and Lake Athabasca. Consequently, concerns have been mounting there about potentially increasing supply of contaminants from oil sands mining activities (Campbell and Spitzer 2007; Wesche 2007, 2009; Aboriginal Affairs and Northern Development Canada (AANDC) and Department of Environment and Natural Resources, Government of the Northwest Territories (ENR-GNWT) 2012). The Slave River and SRD are also part of Environment Canada’s “Phase 2” Integrated Monitoring Plan for the Oil Sands in recognition of the possibility for far-field transport and accumulation of oil sands contaminants (Environment Canada 2011).

Here, we report the results from PAC analyses of a sediment core obtained from a flood-prone lake in the SRD. Using chronological and geochemical methods similar to those employed previously at the site (Brock et al. 2010), we first reconstructed past hydrological conditions and distinguished intervals of high flood influence from intervals of low flood influence. Then, we measured the concentration of PACs in the sediment samples to determine if river-transported bitumen-associated indicator PACs are detectible within the sediment core, and if so, how the concentration and proportion of these compare to those found in PAD31 (Hall et al. 2012) and how they vary over time and between different hydrological conditions.

## Materials and methods

### Site description

The SRD is a large floodplain consisting of numerous river channels, wetlands, shallow lakes, and forests and provides important habitat for a diverse array of wildlife including muskrat, migratory waterfowl, and large mammals (Fig. 1; English et al. 1997; Brock et al. 2010). Lakes span broad hydrological gradients due to differences in the relative influence of snowmelt runoff, rainfall, river flooding, seiche events from Great Slave Lake, and evaporation (Brock et al. 2007). The SRD is accessed by residents of the town of Fort Resolution, NWT, who utilize natural resources in the delta for traditional lifestyle activities (Wolfe et al. 2007).

The SRD has formed as a result of deposition of sediments carried by the Slave River (Fig. 1). The Slave River begins just north of the PAD at the confluence of the Peace River and Rivière des Rochers. Peace River discharge contributes 66 % of the annual average flow to the Slave River (English et al. 1997). The remaining flow (34 %) originates from multiple sources including the Athabasca River and Lake Athabasca, which drain northwards via the Rivière des Rochers, Revillon Coupé, and Chenal des Quatre Fourches.

The study site, SD2 (61° 16′ 56.21″ N, 113° 34′ 55.51″ W), is a small (∼1.2 km^2^), shallow (maximum depth ∼1.5 m) flood-dominated lake in the SRD (Brock et al. 2010). SD2 was chosen to conduct this study for two primary reasons. First, the lake is situated directly adjacent to where the Slave River bifurcates to the Resdelta Channel and other distributaries at the apex of the SRD (Fig. 1). Here, a tight meander bend reroutes ∼90 % of the Slave River flow via the Resdelta Channel (English et al. 1997), and thus, the lake occupies an area where ice jam flooding is known to occur. As a result, SD2 is highly susceptible to river flooding and well-situated to record changes over time in materials transported to the SRD by the Slave River. Second, a previous study by Brock et al. (2010) established a detailed flood record for SD2 dating back to 1924 based on analysis of a sediment core obtained in 2004, which enables the assessment of sediment-borne contaminants transported by the Slave River.

### Sediment core collection

On September 16, 2011, a 48-cm-long sediment core was collected from SD2 using a Glew (1989) gravity corer fitted with a Lucite tube (7.62 cm inner diameter) and was transported to Deninu School in Fort Resolution, where the core was sectioned at 1-cm stratigraphic intervals using a vertical extruder (Glew 1988). This subsampling was coarser than the 0.5-cm stratigraphic sampling intervals used by Brock et al. (2010) to ensure sufficient sample mass for PAC (this study) and metal (MacDonald et al. 2016) analyses. Each sediment sample from the newly obtained sediment core was sealed in a Whirl-Pak® bag and stored in the dark at 4 °C for no more than a few months prior to analyses.

### Lake sediment core analyses

#### Sediment core chronology

As reported in Brock et al. (2010) for the previously analyzed sediment core from SD2, ^210^Pb dating methods were ineffective because rapid sedimentation rates resulted in low ^210^Pb activity even in the upper strata. Instead, ^137^Cs activity was used to develop the sediment core chronology. Based on the correlation of stratigraphic changes in geochemical variables between our sediment core and the one reported in Brock et al. (2010), the ^137^Cs peak was anticipated to occur between 23 and 34 cm depth in the core from 2011. Contiguous sediment samples were prepared and analyzed for ^137^Cs activity from this portion of the sediment core. Additional samples spanning the core (every fourth interval) were also prepared and analyzed for ^137^Cs. Approximately 5–6 g of freeze-dried sediment per sample was packed into plastic tubes to a height of 35 mm, and a silicone septum (Supelco®) was placed on top of the sediment, followed by 1 cm^3^ of 2-ton epoxy resin (Devcon® product no. 14310). ^137^Cs activity was measured to locate the 1963 peak of maximum atmospheric fallout (Appleby 2001). The ^137^Cs peak was used to calculate an average sedimentation rate from 1963 to the top of the core and was extrapolated linearly to the bottom of the core, in the same manner as employed by Brock et al. (2010).

#### Physical and geochemical analyses

Organic matter content was determined from each 1-cm interval by loss on ignition at 550 °C for 2 h, following methods of Heiri et al. (2001). For analysis of organic carbon and nitrogen elemental and isotope composition, 1–2 g of dry sediment from each 1-cm interval was treated with 10 % HCl to remove carbonate constituents. The samples were then rinsed with deionized water to remove the HCl, frozen and freeze-dried, and then sieved (500-μm mesh) to remove the coarse fraction. A measured mass of the remaining fine fraction was submitted to the University of Waterloo Environmental Isotope Laboratory for analysis using an elemental analyzer interfaced with a continuous flow isotope ratio mass spectrometer. Stable carbon isotope results are expressed as δ values in per mil (‰) relative to the Vienna-PeeDee Belemnite (VPDB) standard, and nitrogen stable isotope results are expressed as δ values in ‰ relative to the atmospheric standard (AIR). Percent dry weight organic carbon and nitrogen contents were used to calculate carbon-to-nitrogen (C/N) ratios. Analytical uncertainties for elemental organic carbon and nitrogen content are ±0.21 and ±0.02 %, respectively, while uncertainties for δ^13^C_org_ and δ^15^N are ±0.10 and ±0.21 ‰, respectively, based on sample replicates.

In the study by Brock et al. (2010), physical and geochemical results from a flood deposit obtained in the catchment of SD2 following a spring flood event in 2005 were used to identify flood deposits in their sediment core. Here, we also compare results from the newly acquired sediment core to the 2005 flood deposit to designate stratigraphic intervals of high flood influence.

#### PAC analyses

For analysis of the composition and concentration of PACs, subsamples of 4–10 g (mean = 6.44 g) of wet sediment from each 1-cm interval of the sediment core were placed into amber glass jars with Teflon lid liners that were pre-washed with a 5 % HCl solution and then rinsed with a solvent (dichloromethane). Samples were kept in a cooler (at approximately 4 °C) during express shipment to ALS Environmental Ltd. in Edmonton for analysis of 52 PACs and alkylated PACs using the US Environmental Protection Agency method EPA 3540/8270-GC/MS. Based on analysis of replicate samples, mean precision of individual PAC concentrations was ±7.4 %.

A full set of samples from the entire length of the sediment core (0–48 cm) was originally submitted with ∼4.5 g of dry-mass-equivalent wet sediment. After receiving results of PAC concentrations from the analytical lab, we discovered that three of the seven river-transported bitumen-associated indicator PACs (C2–C4 fluoranthenes/pyrenes) occurred at concentrations below the analytical detection limit in 21 of the samples throughout the core. In an attempt to improve the detection limits for these 21 samples, larger masses of subsamples (∼10 g of dry-mass-equivalent wet sediment) were resubmitted for analysis of PAC concentrations. Results of these resubmitted samples achieved lower detection limits, and consequently, they indicated a consistent presence and quantification of the seven river-transported bitumen-associated indicator PACs. Moreover, measureable concentrations were determined in the resubmitted samples for 46 of the 52 PACs measured by the lab, compared to 36 of 52 PACs detected in the original submissions. Results for the individual PACs are expressed as concentrations (mg g^−1^ dry sediment mass) and as relative proportion of all PACs present in a sample.

### Numerical analyses

We performed one-way analysis of similarity (ANOSIM) tests on the PAC data, expressed as relative proportions, to determine if the composition of PACs differs between groups of sediment samples. One ANOSIM test was performed on the 21 depth intervals that were resubmitted for analysis with greater sample mass and the same depth intervals with results prior to resubmission to determine if the composition of PACs differs due to the mass of sediment analyzed. This test determined if we could combine data from the original submission and the resubmission with larger sediment mass in subsequent data analyses or not. Based on the paleohydrological analyses, 14 of the resubmitted sample intervals were characterized as having been deposited during periods of high flood influence. Eight of these were deposited prior to oil sands development (1931, 1937–1950), and six were deposited after oil sands development began (1986–1994, 1999). A second one-way ANOSIM test was conducted on these 14 resubmitted samples to determine if the composition of PACs has changed pre- versus post-oil sands development. Following the second ANOSIM test, a similarity of percentage (SIMPER) analysis was conducted on the relative proportions of all detected PACs in the same 14 samples to determine which of the 46 detected PACs best discriminated between pre- and post-oil sands development sediments. ANOSIM and SIMPER analyses were performed using the software PRIMER version 6.1.5 (Clarke and Warwick 2001; Clarke and Gorley 2006) and followed methods detailed in Hall et al. (2012).

We conducted three different independent-samples *t* tests to determine if concentrations of PACs differ between samples representing pre- (∼1931, 1937–1950) and post-oil sands development (∼1986–1994, 1999). The first test compared if the total concentration of all PACs differs between sediments deposited pre- versus post-oil sands development when SD2 was strongly influenced by flooding. The other two independent-samples *t* tests were conducted on the subset of PACs identified by Hall et al. (2012) as river-transported bitumen-associated indicator PACs. One test compared the summed *concentration* of these indicator PACs between the two intervals of high flood influence to determine if the mean concentration differs between flood-sourced sediments deposited before versus after development of oil sands. The other tests compared the sum of the *relative proportions* of these indicator PACs between the two intervals of high flood influence to determine if the mean proportion differs between flood-sourced sediments deposited before versus after development of oil sands. The independent-samples *t* tests were performed using the software IBM SPSS Statistics version 20.0.

## Results and interpretation

### Physical core description

The sediment core contained occasional dark gray beds and laminations, ranging from 1 to 4 cm in thickness, and was otherwise light gray. A prominent dark gray bed occurred from 30 to 34 cm depth.

### Sediment core chronology

**Fig. 2:**
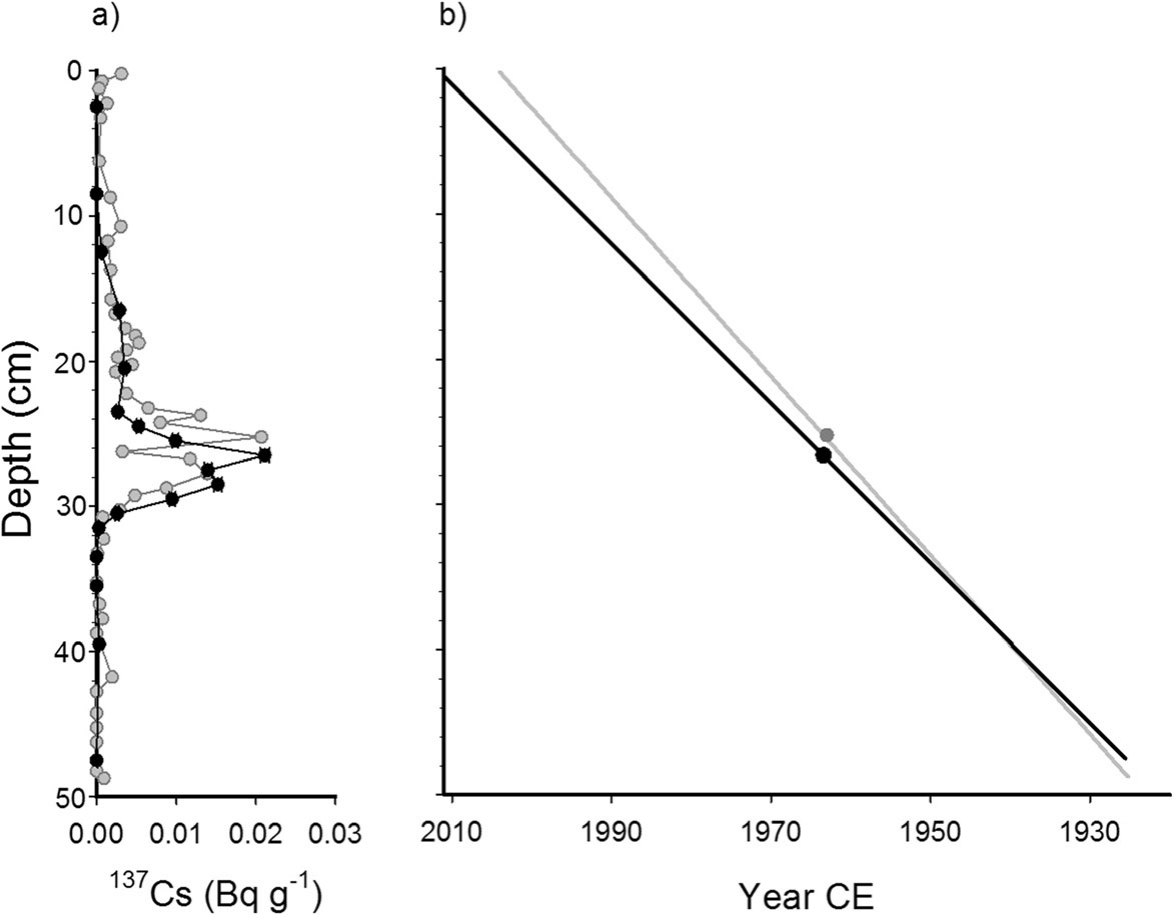
*a* ^137^Cs activity profile and *b* estimated age-depth relationship for the sediment core collected in 2004 by Brock et al. (2010) (*gray symbols* and *solid gray lines*) and the core collected in 2011 for the analysis of PACs (*black symbols* and *solid black lines*)

Brock et al. (2010) identified a ^137^Cs activity peak (0.021 Bq g^−1^ dry sediment) at 25.5 cm depth in the sediment core that was collected in 2004 (Fig. 2a). In the sediment core we collected in 2011, measurements identified a zone of elevated ^137^Cs activity between 24 and 30 cm depth, with an identical peak value (0.021 Bq g^−1^) centered at 26.5 cm depth. Not surprisingly, the ^137^Cs maximum is slightly deeper in the core collected in 2011 than the one collected in 2004, because sediments have accumulated in the lake since 2004. Based on the assumption that the peak in ^137^Cs activity represents 1963, the year of maximum atmospheric fallout from aboveground nuclear weapons testing (Appleby 2001), the average sedimentation rate is 0.54 cm year^−1^ to the top of the core. This value is comparable to, though slightly lower than, the average sedimentation rate of 0.62 cm year^−1^ reported in Brock et al. (2010). A small difference in sedimentation rates may be explained by the fact that the newer sediment core was obtained from a more distal location to where Slave River floodwaters enter the basin. Using the same approach as Brock et al. (2010), this sedimentation rate was extrapolated to the bottom of the core, which generated an estimated basal date of 1924 (Fig. 2b).

### Designation of high flood influence intervals

**Fig. 3:**
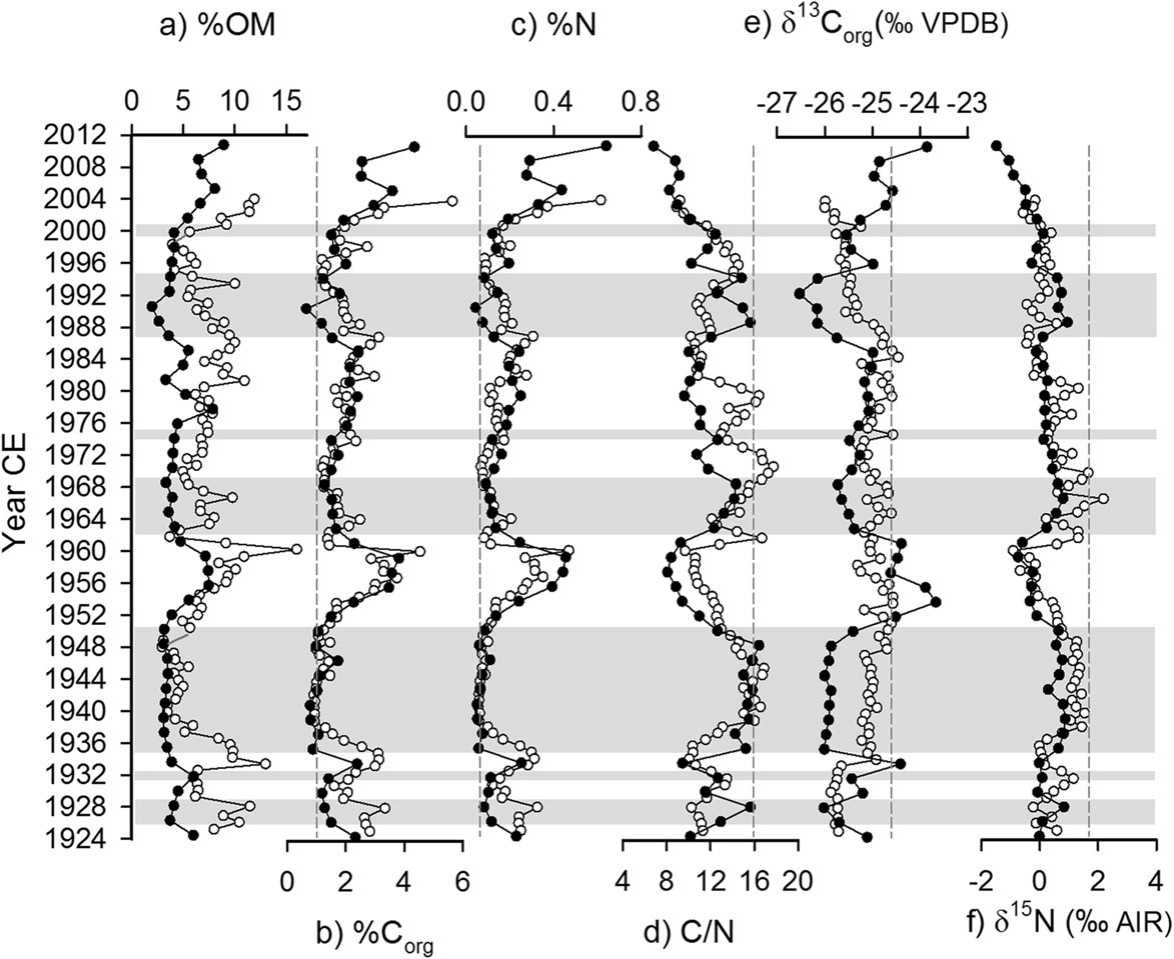
Stratigraphic profiles showing variations in physical and geochemical variables in sediment cores from Brock et al. (2010; *white symbols*) and this study (*black symbols*): *a* organic matter content as a percentage of dry mass (*%OM*), *b* organic carbon content as a percent of dry mass (*%C*_*org*_), *c* nitrogen content as a percent of dry mass (*%N*), *d* carbon to nitrogen (*C/N*) ratio, *e* δ^13^C_org_, and *f* δ^15^N. The *vertical dashed lines* in *b*–*f* represent the composition of the Slave River sediment collected from the land surface within the catchment of SD2 shortly after a spring flood event in 2005 (Brock et al. 2010). *Gray horizontal bars* indicate interpreted intervals of high flood influence based mainly on the C/N ratio presented in *d*

Physical and geochemical results for the sediment core collected in 2011 showed distinct variations in the content of organic matter (%OM), organic carbon (%C_org_), total nitrogen (%N), C/N ratio, and isotope compositions of organic carbon (δ^13^C_org_) and nitrogen (δ^15^N) (Fig. 3). The stratigraphic patterns of variation aligned closely with those reported in the core analyzed by Brock et al. (2010) (Fig. 3), especially for %C_org_ and %N. Values also often approached the geochemical signatures of the flood deposit obtained in 2005 (Brock et al. 2010). These intervals are characterized by high values of C/N and δ^15^N and low values of %OM, %C_org_, and %N. Shifts to low OM, %C_org_, %N, and δ^13^C_org_ are consistent with a decline in lake productivity and influx of mineral matter to lakes. And high values of sedimentary C/N are consistent with an influx of terrestrial organic matter supplied by floodwaters. These intervals corresponded with light gray bedding evident in the sediment core. In accordance with results from Brock et al. (2010), C/N results appear to be the most sensitive recorder of river flooding in sediments deposited at SD2, and intervals with C/N ≥ 12 were interpreted as deposited during high flood influence (dated at ∼1926–1928, ∼1931, ∼1935–1950, ∼1963–1968, ∼1974, ∼1986–1994, and ∼1999; Fig. 3, gray bars). Conversely, high %OM, %C_org_, %N, and δ^13^C_org_ and low δ^15^N define intervals of low flood influence. A period of particularly high OM, %C_org_, %N, and δ^13^C_org_ and low C/N and δ^15^N was identified at ∼1952–1961 indicating the lowest flood influence in the sediment record, which corresponded to the prominent dark gray bed at 30–34 cm depth. Other intervals of low flood influence include ∼1975–1985 and ∼2001–2011.

Results for %OM, C/N, and δ^15^N showed subtle differences in the timing and magnitude of some peak values between the two sediment cores collected in 2004 and 2011, and δ^13^C_org_ was quite different between the two cores. In Brock et al. (2010), reported intervals of high flood influence included ∼1931–1933, ∼1938–1948, ∼1963, ∼1968–1974, ∼1976–1981, and ∼1992–1998. Absent from the newly obtained sediment core is the interval of high flood influence from ∼1976 to 1981 identified by Brock et al. (2010). Also undetectable in the new sediment core is the 2005 flood event. This feature and minor inconsistencies in the reporting of high flood influence intervals between the two cores may be a consequence of a coarser 1.0-cm sampling interval compared to the 0.5-cm sampling interval employed by Brock et al. (2010), small differences and uncertainties in the sedimentation rates and core chronologies, and more distal location of the new core with respect to where floodwaters enter the basin.

### PACs

#### Stratigraphic results

**Fig. 4:**
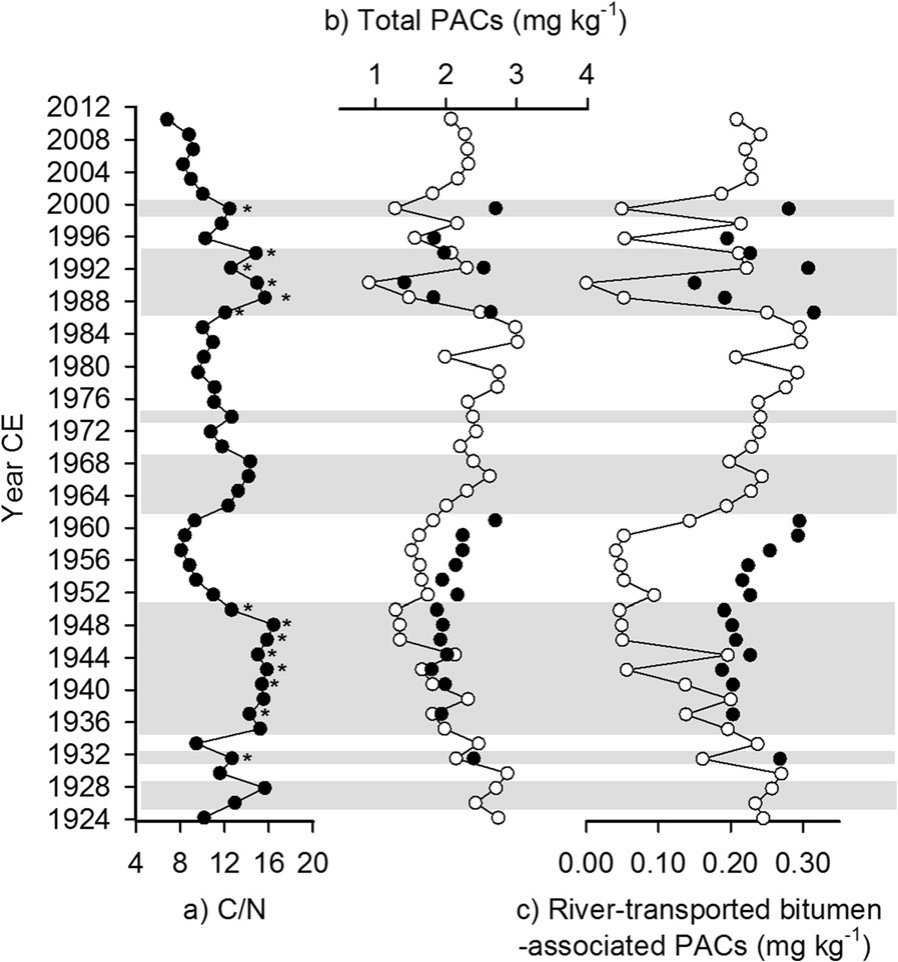
*a* C/N ratio, *b* concentration of total PACs, and *c* concentration of river-transported bitumen-associated indicator PACs versus time for both original samples (*white symbols*) and samples resubmitted with higher sediment mass (*black symbols*) . *Horizontal gray bars* represent intervals of high flood influence (refer to Fig. 3). *Asterisks* in *a* represent the 14 resubmitted samples that fall within intervals of high flood influence

Concentrations of total PACs varied by more than threefold throughout the sediment core, ranging from 0.906 to 3.011 mg kg^−1^ (mean = 2.086 ± 0.491 mg kg^−1^ (1 SD)) (Fig. 4b). Concentrations of river-transported bitumen-associated indicator PACs also varied throughout the sediment core and ranged from below detection limit (0.04 mg kg^−1^; assigned value as 0) to 0.297 mg kg^−1^ (mean = 0.176 ± 0.086 mg kg^−1^) (Fig. 4c). Temporal variations in concentrations of total PACs and river-transported bitumen-associated indicator PACs illustrate that high flood influence intervals possessed generally lower PAC concentrations than intervals of low flood influence (Fig. 4a–c).

As a result of resubmitting 21 samples with a higher mass for PAC analysis, the total concentration of PACs for these 21 samples increased by ∼12 % on average (SD = 9 %, min = −2.8 %, max = 35.9 %) compared to values obtained from the original submission with smaller sediment mass (Fig. 4b). And resubmissions accounted for an average increase of 46.1 % in river-transported bitumen-associated indicator PACs (SD = 26.2 %, min = 3.7 %, max = 100 %; Fig. 4c). An ANOSIM test indicated that composition of PACs differs significantly between the original and the resubmitted samples with higher sediment mass (*R* statistic = 0.895, *P* < 0.001, 9999 permutations performed). Thus, differences in detection limits and concentrations of PACs between the original submission and resubmission made it impossible to consistently interpret patterns throughout the entire sediment core, but the resubmitted samples have provided results of higher quality for 21 samples, 14 of which are within intervals of high flood influence (Fig. 4a, refer to asterisks) and seven are within intervals of low flood influence. Of the 14 samples within intervals of high flood influence, eight were deposited prior to oil sands development (∼1931, 1937–1950) and six were deposited after (∼1986–1994, 1999), hereafter referred to as pre- and post-oil sands samples. Below, concentrations and composition of PACs (including the seven river-transported bitumen-associated indicator PACs) are examined for these samples and are then compared to previously obtained results from PAD31 in the Athabasca Delta (Hall et al. 2012).

#### Pre- versus post-oil sands development

The total PAC concentration averaged 1.982 ± 0.026 mg kg^−1^ in pre-oil sands samples and 2.177 ± 0.524 mg kg^−1^ in post-oil sands samples, and the concentration of river-transported bitumen-associated indicator PACs averaged 0.211 ± 0.026 mg kg^−1^ for pre-oil sands samples and 0.245 ± 0.055 mg kg^−1^ for post-oil sands samples (Table 1). Independent-samples *t* tests indicated no significant difference in total PAC concentration (*t* = 0.878, *P* = 0.414, *d.f.* = 5.886) or river-transported bitumen-associated indicator PAC concentration (*t* = 1.188, *P* = 0.279, *d.f.* = 6.136) between pre- and post-oil sands samples.

**Table 1:** Total PAC concentration and total concentration and proportion of the seven river-transported bitumen-associated indicator PACs for the 14 resubmitted samples deposited during high flood-influence intervals

	Sediment core depth interval (cm)	Estimated sample date	Total PACs (mg kg^−1^)	Total river-transported bitumen-associated indicator PACs (mg kg^−1^)	Proportion of river-transported bitumen-associated indicator PACs
Post-oil sand samples	6–7	2000	2.701	0.280	0.104
	9–10	1994	1.970	0.227	0.115
	10–11	1992	2.533	0.207	0.121
	11–12	1991	1.407	0.150	0.107
	12–13	1989	1.820	0.192	0.105
	13–14	1987	2.633	0.315	0.120
Average			2.177 ± 0.524	0.245 ± 0.055	0.112 ± 0.008
Pre-oil sand samples	33–34	1951	1.868	0.191	0.102
	34–35	1949	1.952	0.202	0.103
	35–36	1947	1.919	0.207	0.108
	36–37	1945	2.012	0.227	0.113
	37–38	1943	1.793	0.188	0.105
	38–39	1941	1.984	0.203	0.102
	40–41	1938	1.933	0.203	0.105
	43–44	1932	2.391	0.268	0.112
Average			1.982 ± 0.026	0.211 ± 0.026	0.106 ± 0.004

**Fig. 5:**
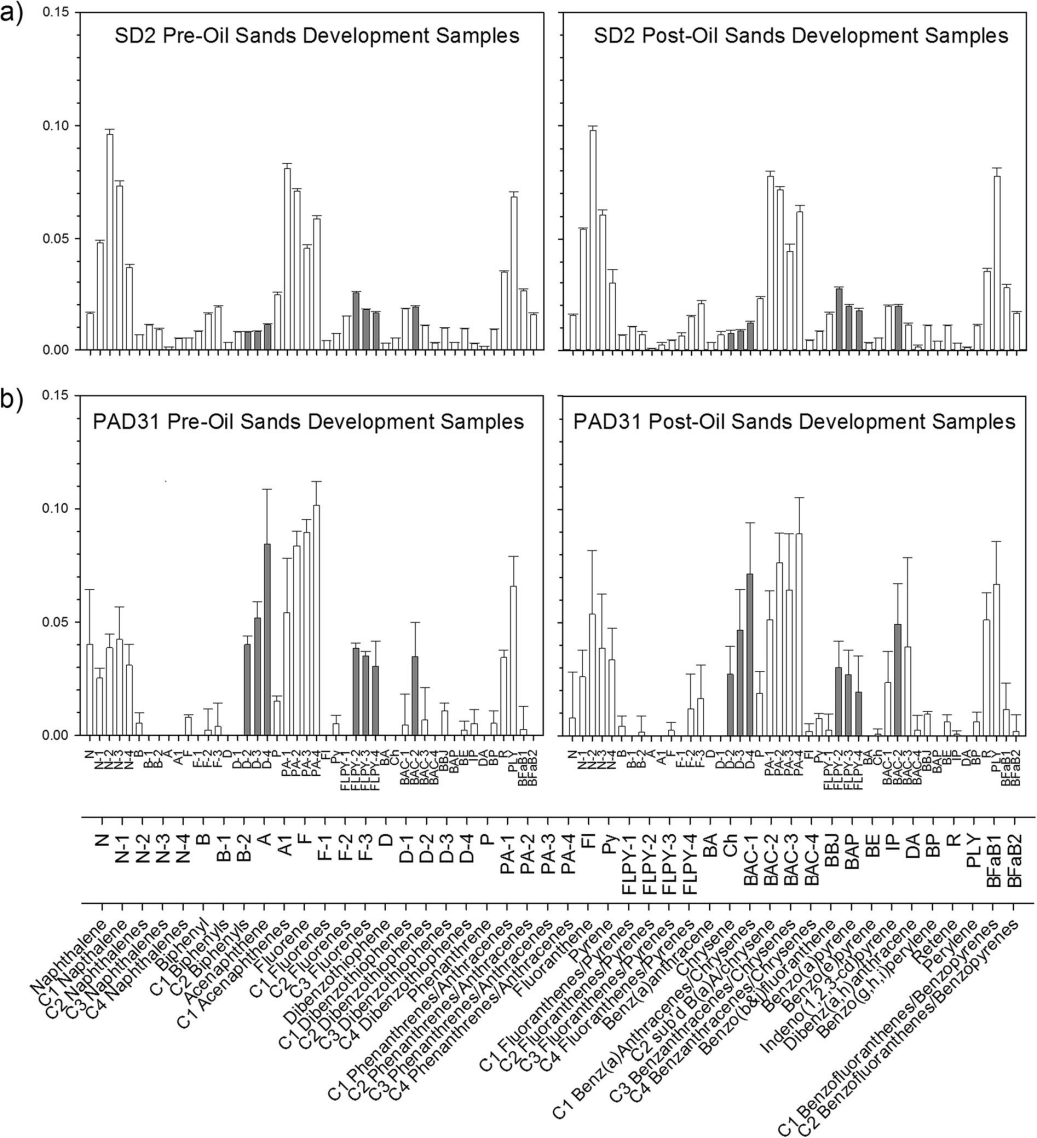
Average proportions of 46 detected PACs for the resubmitted samples representing periods of high flood influence pre- versus post-oil sands development for *a* SD2 and *b* PAD31. *Error bars* represent the standard error of individual PACs about the sample average. *Gray bars* indicate the seven river-transported bitumen-associated indicator PACs identified by Hall et al. (2012)

At SD2, PACs were dominated by alkylated naphthalenes, phenanthrenes/anthracenes, and perylene in both pre- and post-oil sands samples (Fig. 5a). An ANOSIM test identified a significant difference in the proportions of the 46 detected PACs between pre- and post-oil sands samples (*R* statistic = 0.265, *P* = 0.013). However, a SIMPER analysis detected only 6.4 % dissimilarity in PAC proportions between pre- and post-oil sands samples. PACs accounting for most of the dissimilarity were not those identified as river-transported bitumen-associated indicator PACs by Hall et al. (2012) (Table 2). River-transported bitumen-associated indicator PACs contributed only 0.68 % of the total of 6.4 % of total dissimilarity or ∼10 % of total dissimilarity between pre- and post-oil sands samples. Overall, PAC composition is similar between pre- and post-oil sands samples (93.6 % similarity) and differences in relative proportions of individual PACs are subtle (Table 2). This includes river-transported bitumen-associated PACs, for which the average proportion was 0.106 ± 0.004 in pre-oil sands samples and 0.112 ± 0.008 in post-oil sands samples (Table 1). Independent-samples *t* test indicated no significant difference in mean proportions of river-transported bitumen-associated indicator PACs between pre- and post-oil sands samples (*t* = 1.627, *P* = 0.146, *d.f.* = 7.221).

**Table 2:** Results of similarity of percentage (SIMPER) analysis on proportions of the 46 measured PACs in the pre- versus post-oil sands high flood influence intervals. Asterisks are used to denote the seven river-transported bitumen-associated indicator PACs identified by Hall et al. (2012)

	Average % composition (pre-oil sands)	Average % composition (post-oil sands)	Average dissimilarity (%)	Contribution to total dissimilarity (%)
Fluorene	0.53	0.44	0.05	0.70
C1 napthalene	4.81	5.43	0.31	4.80
C2 naphthalenes	9.63	9.79	0.30	4.64
C3 naphthalenes	7.35	6.05	0.65	10.02
C4 naphthalenes	3.66	2.97	0.47	7.28
Biphenyl	0.68	0.66	0.02	0.38
C1 biphenyls	1.10	1.04	0.04	0.66
C2 biphenyls	0.92	0.70	0.12	1.88
Acenaphthene	0.12	0.07	0.04	0.59
C1 acenaphthenes	0.52	0.24	0.15	2.28
Fluorene	0.53	0.44	0.05	0.70
C1 fluorenes	0.83	0.64	0.12	1.78
C2 fluorenes	1.60	1.47	0.10	1.48
C3 fluorenes	1.91	2.07	0.15	2.28
Dibenzothiophene	0.34	0.34	0.02	0.26
C1 dibenzothiophenes	0.81	0.69	0.10	1.54
C2 dibenzothiophenes*	0.80	0.75	0.11	1.74
C3 dibenzothiophenes*	0.81	0.88	0.05	0.75
C4 dibenzothiophenes*	1.13	1.21	0.09	1.34
Phenanthrene	2.44	2.29	0.16	2.54
C1 phenanthrenes/anthracenes	8.11	7.77	0.32	5.00
C2 phenanthrenes/anthracenes	7.10	7.18	0.17	2.63
C3 phenanthrenes/anthracenes	4.58	4.47	0.32	4.95
C4 phenanthrenes/anthracenes	5.88	6.19	0.36	5.55
Fluoranthene	0.42	0.44	0.03	0.47
Pyrene	0.74	0.83	0.05	0.72
C1 fluoranthenes/pyrenes	1.50	1.61	0.08	1.17
C2 fluoranthenes/pyrenes*	2.54	2.71	0.11	1.69
C3 fluoranthenes/pyrenes*	1.80	1.95	0.09	1.38
C4 fluoranthenes/pyrenes*	1.64	1.75	0.15	2.28
Benz(a)anthracene	0.32	0.33	0.01	0.19
Chrysene	0.53	0.54	0.02	0.34
C1 benz(a)anthracenes/chrysenes	1.82	1.95	0.08	1.30
C2 sub’d B(a)A/chrysene*	1.91	1.96	0.08	1.29
C3 benzanthracenes/chrysenes	1.07	1.13	0.09	1.40
C4 benzanthracenes/chrysenes	0.32	0.16	0.09	1.46
Benzo(b and j)fluoranthene	0.98	1.08	0.06	0.88
Benzo(a)pyrene	0.33	0.39	0.03	0.50
Benzo(e)pyrene	0.94	1.09	0.07	1.13
Indeno(1,2,3-cd)pyrene	0.29	0.32	0.02	0.32
Dibenz(a,h)anthracene	0.16	0.11	0.03	0.49
Benzo(g,h,i)perylene	0.92	1.09	0.10	1.47
Retene	3.45	3.50	0.15	2.25
Perylene	6.86	7.77	0.54	8.36
C1 benzofluoranthenes/benzopyrenes	2.61	2.76	0.18	2.72
C2 benzofluoranthenes/benzopyrenes	1.57	1.64	0.11	1.74
Sum of river-transported bitumen-associated indicator PACs	11.21	10.63	0.68	10.47

#### SD2 versus PAD31

**Fig. 6:**
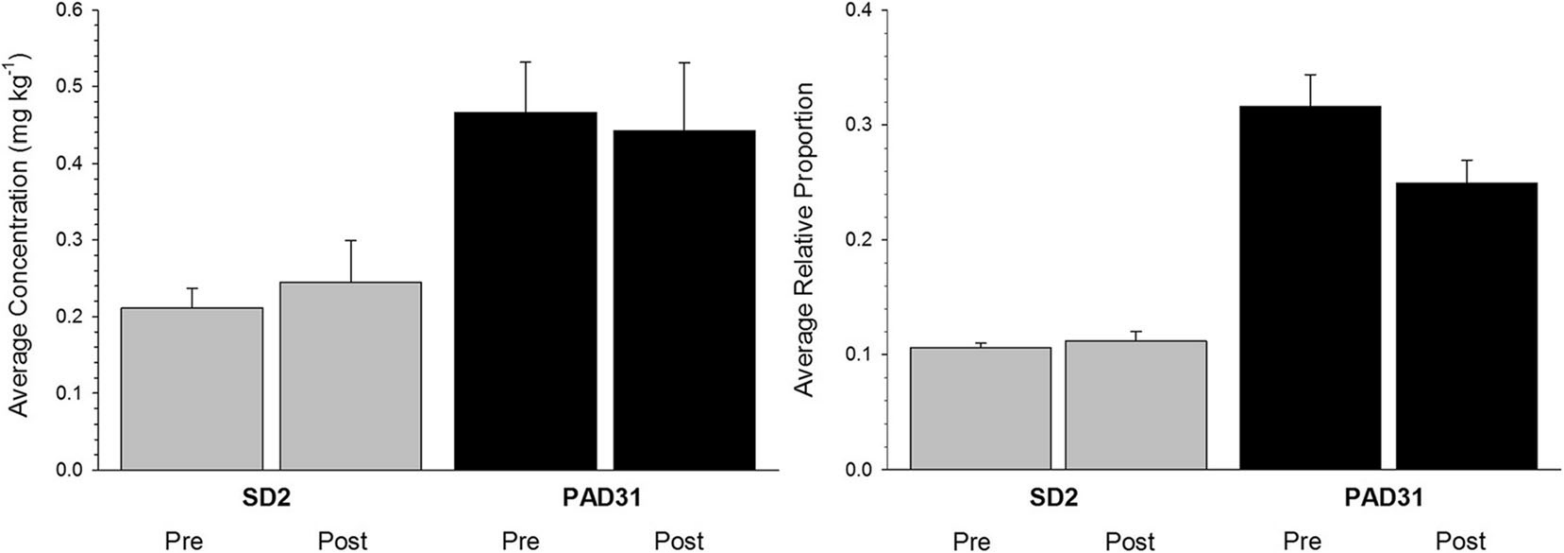
A comparison of average concentration and relative proportion of river-transported bitumen-associated PACs in SD2 (*gray bars*) and PAD31 (*black bars*) sediments deposited during intervals of high flood influence pre- versus post-oil sands development. *Error bars* represent 1 standard deviation about the sample average

For sediments deposited during high flood influence intervals, average concentrations and proportions of river-transported bitumen-associated indicator PACs in both pre- and post-oil sands samples at SD2 were much lower than those obtained at PAD31 (Fig. 6). For example, average concentrations from 1986 to 1994 and 1999 at SD2 (0.245 ± 0.055 mg kg^−1^ (1 SD)) were 45 % lower than those at PAD31 (0.442 ± 0.089 mg kg^−1^) during roughly the same time interval (1987–1999). Similarly, average relative proportions of each river-transported bitumen-associated indicator PAC were much lower at SD2 (pre-oil sands = 0.106 ± 0.004, post-oil sands = 0.112 ± 0.008) than at PAD31 (pre-oil sands = 0.315 ± 0.028, post-oil sands = 0.249 ± 0.020) (Figs. 5(b) and 6).

**Fig. 7:**
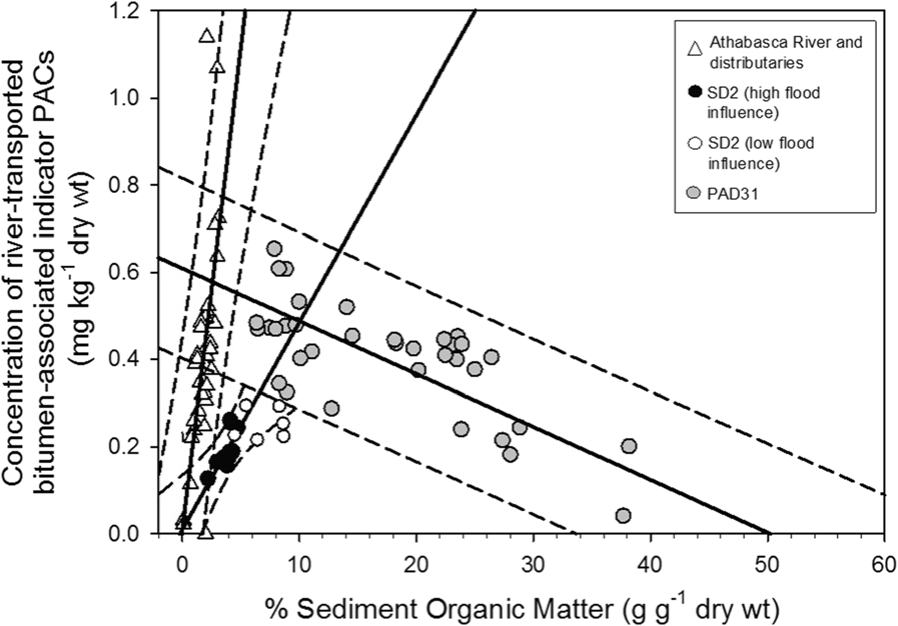
Linear relations (*black lines*) with 95 % prediction intervals (*dashed lines*) between river-transported bitumen-associated indicator PACs and organic matter content in bottom sediments from the downstream Athabasca River and its distributaries (*white triangles*; Regional Aquatics Monitoring Program, obtained online: http://www.ramp-alberta.org/ramp/data.aspx), and *lake sediments* from SD2 (*black circles* for high flood influence samples; *white circles* for low flood influence samples) and PAD31 (*gray circles*; Hall et al. 2012). Note that the linear relation shown for PAD31 is also based on data from PAD23 (not shown; see Hall et al. 2012)

The total concentration of river-transported bitumen-associated indicator PACs from pre- and post-oil sands samples was plotted versus organic matter content to explore further processes responsible for their deposition at SD2 (Fig. 7). Results identified a positive relation for sediments deposited at SD2 during intervals of high flood influence, similar to the relation for the Athabasca River and distributaries, but with a lower slope at SD2. Sediments deposited during intervals of low flood influence at SD2 also mostly plotted along this same positive relation. The relation for SD2 contrasts with the negative slope previously obtained for PAD31 (and “PAD23” (not shown); Hall et al. 2012).

## Discussion

### Deposition of river-transported bitumen-associated indicator PACs at SD2

In the absence of long-term river monitoring data, paleolimnological methods applied to floodplain lakes provide a means for generating baseline knowledge of river contaminant concentrations and composition, because these lakes can archive environmental information prior to human disturbance. Analysis of a sediment core from flood-dominated lake SD2, located at the apex of the Slave River Delta, revealed that river-transported bitumen-association indicator PACs were deposited in the SRD well before the onset of oil sands development in 1967, but in concentrations much lower than in a floodplain lake in the Athabasca Delta. This finding is consistent with the more distal location of the SRD with respect to the Alberta oil sands. Lower concentrations and proportions of river-transported bitumen-associated indicator PACs are likely a consequence of sediment retention within the Athabasca Delta and Lake Athabasca, plus dilution with sediment from the Peace River, which contributes 66 % of annual average flow to the Slave River (English et al. 1997).

Comparison to the relations between river-transported bitumen-associated indicator PAC concentration and organic matter content for PAD31 reported in Hall et al. (2012) illustrates that these PACs in SD2 sediments are not only diluted by distance from source but also by flood energy conditions (Fig. 7). Hall et al. (2012) identified two distinct trends when comparing river-transported bitumen-associated indicator PAC concentrations and organic matter content in river and lake sediments in the Athabasca Delta. River-transported bitumen-associated indicator PAC concentrations in the sediment record of lakes PAD31 and PAD23 (not shown) were found to be significantly and *negatively* correlated with organic matter content, suggesting that these two lakes underwent periodic inoculation with river-borne sediments that were relatively low in organic matter content and relatively high in river-transported bitumen-associated indicator PAC concentration. Conversely, when these lakes experienced less flooding, sedimentary concentrations of the river-transported bitumen-associated indicator PACs were much lower, which were attributed to dilution from organic matter generated by high in-lake aquatic production.

In contrast to PAD31, river-transported bitumen-associated indicator PACs from SD2 showed a strikingly different and *positive* relation with organic matter (Fig. 7). This relation stems from the greater affinity of PACs to river sediments with higher organic matter content (Hall et al. 2012). The relation at SD2 is similar to that observed in river sediment collected from the Athabasca River and its distributaries (i.e., a positive slope), suggesting that high flood influence samples at SD2 are characteristic of river sediment with little if any influence on PAC content by autochthonous organic matter. Supporting this notion, concentrations of river-transported bitumen-associated indicator PACs in SD2 were slightly higher during an interval of particularly low flood influence (and high organic matter content) between ∼1952 and ∼1961 (0.256 ± 0.037 mg kg^−1^ (1 SD)) compared to intervals of high flood influence pre- and post-oil sands development (Figs. 3 and 4c), but these samples also mostly fall on the same positive relation in Fig. 7, as defined by the high flood influence intervals. Thus, it is likely that river-transported bitumen-associated PACs in SD2 are diluted not only by distance from source but also by high-magnitude flood events in the Slave River, when coarse-grained sediment with low organic content is predominant. These findings demonstrate the need to understand the paleohydrology of floodplain lakes to interpret their contaminant stratigraphic profiles, as we and others have shown (Foster and Charlesworth 1996; Audry et al. 2004; Schulz-Zunkel and Krueger 2009; Hall et al. 2012; MacDonald et al. 2016).

We acknowledge that these results are based on the data obtained from one flood-dominated basin and may not be a representative of the SRD as a whole, but SD2 is situated at a tight meander bend at the junction of the Slave River and Resdelta Channel within the active portion of the delta (Fig. 1). Basins within this area of the delta are known to receive river floodwaters during the spring freshet (Brock et al. 2008), a period when oil sands-associated contaminants become entrained within the Athabasca River and its distributaries and transported downstream (Kelly et al. 2009; Kelly et al. 2010; Giesy et al. 2010; Hall et al. 2012). Thus, SD2 is well-situated to record changes over time in materials transported to the SRD by the Slave River and to compare these materials with those transported by the Athabasca River and deposited in the Athabasca Delta.

### Assessing effects of oil sands development on the SRD

This study did not provide data to address potential industrial contributions of contaminant loading for the past decade, which has been a time of rapid growth of oil sands industry. Nonetheless, we found no significant differences in concentrations and proportions of river-transported bitumen-associated indicator PACs in sediments deposited during periods of high flood influence before (∼1931, 1937–1950) versus after (∼1986–1994, 1999) the onset of oil sands development, consistent with prior results that included more recent flood-derived deposits from the Athabasca Delta (Hall et al. 2012). Furthermore, results showed that river-transported bitumen-associated indicator PACs have been accumulating in SD2 for decades prior to development, just as they have in the Athabasca Delta—a consequence of natural erosion along upstream riverbanks and fluvial entrainment of these contaminants. At SD2, potential natural sources of PACs include seams of coal and bitumen along the Peace River and its tributaries (Jautzy et al. 2015), in addition to exposures of bitumen in the McMurray Formation along the Athabasca River (Hall et al. 2012).

## Conclusions

Baseline knowledge of river contaminant concentrations and composition prior to perturbation is needed to detect pollution and to determine the processes of transport and deposition. We address this critical knowledge gap for river-transported bitumen-associated PAC deposition in the Slave River Delta in response to concerns regarding pollution from the development of the Alberta oil sands 500 km upstream. Analysis of a sediment core (spanning ∼1924–2011) from flood-dominated lake “SD2”, located at the apex of the delta, revealed the deposition of river-transported bitumen-associated indicator PACs decades before oil sands development, owing to natural erosion along upstream riverbanks and fluvial entrainment of these contaminants. Concentrations of these PACs were 45 % lower than in temporally comparable sediments deposited in flood-prone lake PAD31 in the Athabasca Delta due to dilution with respect to distance from source and by high-magnitude flood events in the Slave River that are associated with low organic content. We detected no significant differences in concentrations and proportions of river-transported bitumen-associated PACs in sediments deposited during periods of high flood influence before (∼1931, 1937–1950) and after (∼1986–1994, 1999) oil sands development. While these data suggest that oil sands development has not significantly enhanced the delivery of PACs to SD2 during flood events of the late 1980s and 1990s, the sediment record from SD2 does not contain intervals representing high flood influence during the past decade, a period when oil sands development has increased rapidly. These results, however, are consistent with prior studies that also failed to detect PAC pollution in sediments of a flood-prone lake in the Athabasca Delta (Hall et al. 2012). The new knowledge of baseline concentrations and proportions of river-transported bitumen-associated PACs in SD2 sediments can be used to evaluate ongoing and future river sediment monitoring for evidence of pollution.
